# Effectiveness of an amygdala and insula retraining program combined with mindfulness training to improve the quality of life in patients with long COVID: a randomized controlled trial protocol

**DOI:** 10.1186/s12906-023-04240-0

**Published:** 2023-11-09

**Authors:** Virginia Gasión, Alberto Barceló-Soler, María Beltrán-Ruiz, Rinchen Hijar-Aguinaga, Loreto Camarero-Grados, Yolanda López-del-Hoyo, Javier García-Campayo, Jesus Montero-Marin

**Affiliations:** 1 Aragon Institute for Health Research, IIS Aragon, Zaragoza, Spain; 2 Research Network on Chronicity, Primary Care and Health Promotion, RICAPPS, Zaragoza, RD21/0016/0005 Spain; 3 University of Zaragoza, Huesca, Spain; 4 Department of Psychiatry, University of Oxford, Oxford, UK; 5 Teaching, Research & Innovation Unit, Parc Sanitari Sant Joan de Déu, Sant Boi de Llobregat, Spain; 6 Consortium for Biomedical Research in Epidemiology & Public Health (CIBER Epidemiology and Public Health - CIBERESP), Madrid, Spain

**Keywords:** Long COVID, Amygdala Insula Retraining, Mindfulness training, Quality of life, Randomized Controlled Trial, Psychological interventions

## Abstract

**Background:**

There has been growing clinical awareness in recent years of the long-term physical and psychological consequences of the SARS-CoV-2 virus, known as Long COVID. The prevalence of Long COVID is approximately 10% of those infected by the virus. Long COVID is associated with physical and neuropsychological symptoms, including those related to mental health, psychological wellbeing, and cognition. However, research on psychological interventions is still in its early stages, in which means that available results are still limited. The main objective of this study is to evaluate the effects of a program based on amygdala and insula retraining (AIR) combined with mindfulness training (AIR + Mindfulness) on the improvement of quality of life, psychological well-being, and cognition in patients with Long COVID.

**Methods:**

This study protocol presents a single-blind randomized controlled trial (RCT) that encompasses baseline, post-treatment, and six-month follow-up assessment time points. A total of 100 patients diagnosed with Long COVID by the Spanish National Health Service will be randomly assigned to either AIR + Mindfulness (n = 50) or relaxation intervention (n = 50), the latter as a control group. The primary outcome will be quality of life assessed using the Short Form-36 Health Survey (SF-36). Additional outcomes such as fatigue, pain, anxiety, memory, and sleep quality will also be evaluated. Mixed effects regression models will be used to estimate the effectiveness of the program, and effect size calculations will be made.

**Discussion:**

Long COVID syndrome is a clinical condition characterized by the persistence of symptoms for at least 12 weeks after the onset of COVID-19 that significantly affects people’s quality of life. This will be the first RCT conducted in Spain to apply a psychotherapy program for the management of symptoms derived from Long COVID. Positive results from this RCT may have a significant impact on the clinical context by confirming the beneficial effect of the intervention program being evaluated on improving the symptoms of Long COVID syndrome and aiding the development of better action strategies for these patients.

**Trial registration:**

Clinical Trials.gov NCT05956405. Registered on July 20, 2023.

## Introduction

### Background and rationale

Long COVID, also known as Post-COVID Conditions or SARS-CoV-2 infection with post-acute sequelae, refers to a set of multiple organ symptoms that persist in patients who have experienced SARS-CoV-2 infection, even after the acute phase of the disease [1]. Approximately 10% of individuals experience these symptoms after the resolution of their acute COVID phase [2]. Such symptoms exhibit variability and are multisystemic, and they may be persistent or limited over time. Symptoms consist of fatigue that does not improve with rest, shortness of breath, fever, myalgia, sore throat, chest and back pain, as well as other muscle and mild neuropathic pain. Additionally, symptoms include orthostatic hypotension, intestinal motility issues, symptoms of neural disorders, insomnia that can disrupt sleep and rest, hyperactivation of the sympathetic system, cognitive deficits, anxiety, and the inability to manage stress [3, 4].

Furthermore, it is necessary to acknowledge the psychosocial impact of the miscellaneous symptoms described on the lives of individuals dealing with Long COVID. Limitations in performing everyday activities such as self-care, household tasks, shopping, or engaging in social activities can be constraining, with variation based on symptom severity. Consequently, these limitations generate feelings of isolation and loneliness, which can contribute to secondary depression [5, 6]. All these symptoms can have a significant impact on their physical, occupational, and emotional health, often resulting in social and economic challenges for both individuals and their families [1]. In summary, psychological distress frequently accompanies patients with Long COVID, who often present with symptoms of depression, anxiety, and diminished quality of life, among others [5, 7–10].

Research suggests that Long COVID symptoms are caused by chronic inflammation and immune dysregulation in the body [1]. Recent studies have indicated a potential link between Long COVID and neurological complications, such as cognitive impairment and brain fog [11]. In fact, Long COVID syndrome presents similarly to myalgic encephalomyelitis/chronic fatigue syndrome (ME/CFS) [3], which is characterized by fatigue and immune and cognitive dysfunctions [8]. Due to their similarity, there is reason to believe that ME/CFS treatments could be effective in resolving Long COVID. Emerging evidence suggests that the impact of Long COVID extends beyond physical health, with a significant proportion of patients reporting challenges in their professional and social lives [12]. This underscores the complex nature of the condition and highlights the need for comprehensive treatment approaches and the importance of addressing the holistic wellbeing of individuals affected by this condition.

Psychological approaches, including mind-body therapies such as mindfulness training, have demonstrated their effectiveness for the treatment of these types of pathologies by improving mental health and physical function, while reinforcing acceptance and reducing symptoms [13–15]. Amygdala and insula retraining (AIR) techniques were initially developed for CFS patients as a method to reduce the chronic over-sensitization and heightened fear response of the amygdala, which may underlie certain symptoms associated with both CFS and fibromyalgia [14–16]. These techniques aim to retrain conditioned somatic signaling in the brain that can maintain the nervous and immune systems in a constant state of alert. This retraining is achieved by means of a specialized intervention designed to strengthen neurological inhibitory mechanisms in areas of the prefrontal and orbital cortices, the insula, and the anterior and posterior cingulate [17–20]. AIR techniques involve repetitively interrupting signals from the amygdala and insula using a variety of personalized practices for each patient [18, 20]. In essence, patients learn to recognize internal symptom signals and then take action to disrupt the signaling. Additionally, mindfulness training has been shown to reduce amygdala reactivity and increase gray matter volume in the prefrontal cortex and insula [21]. Consequently, the prospect of combining mindfulness training with AIR techniques to enhance effects is appealing. AIR training has demonstrated efficacy in treating chronic conditions such as ME/CFS [14, 15], and the combined approach of AIR techniques with mindfulness training (AIR + Mindfulness) has proven effective in managing fibromyalgia symptoms [17, 20–22].

However, despite the previously reported positive results, there is still no evidence regarding the possible mediating variables associated with the AIR + Mindfulness program since it is a relatively new psychotherapeutic strategy. Despite this, and based on the results obtained in previous studies, psychological variables such as mindfulness skills, emotion regulation, and experiential avoidance could play a mediating role in the improvement of Long COVID syndrome through the application of the AIR + Mindfulness program [22–24]. For example, a recent observational study identified a relationship suggesting that experiential avoidance is associated with the greater severity of fatigue and pain perceived by patients with COVID-19, and that psychological interventions aimed at working on this psychological variable would favor an improvement in the severity of symptoms in these patients [23]. On the other hand, a longitudinal study that investigated the role of emotion regulation on mental health found that people who used maladaptive emotion regulation strategies reported more symptoms of anxiety, depression, poorer sleep quality, and less life satisfaction [24]. For this reason, psychological interventions aimed at promoting the learning of adaptive regulation strategies (i.e., integrative emotion regulation [25] could be expected to have a positive impact on the improvement and management of symptoms associated with COVID-19 and Long COVID.

Extensive research is being conducted to test pharmaceutical, biological, dietary, homeopathic, and rehabilitative remedies for the treatment of Long COVID symptoms. However, an effective intervention strategy still remains elusive [26, 27]. Recent evidence suggests that psychological interventions can improve mental health and alleviate a variety of symptoms for Long COVID patients [28, 29].

### Objectives and hypotheses

The primary aim of the study proposed in this protocol is to evaluate the impact of the AIR + Mindfulness on enhancing the quality of life of patients with Long COVID. The secondary objectives include (a) to examine the effects of AIR + Mindfulness on pain, pain catastrophizing, fatigue, insomnia, anxiety, depression, and memory symptoms; and (b) to analyze the role of mindfulness skills, emotion regulation, and experiential avoidance as potential mechanisms through which AIR + Mindfulness exerts its effects.

The main hypothesis is that AIR + Mindfulness will be more effective than relaxation therapy for improving quality of life among patients diagnosed with Long COVID at post-treatment. The secondary hypotheses are (a) AIR + Mindfulness will be more effective than relaxation therapy for improving pain, pain catastrophizing, fatigue, insomnia, anxiety, depression, and memory symptoms at post-treatment; (b) improvements in all psychological variables will be sustained at the three-month follow-up; and (c) mindfulness skills, emotion regulation, and experiential avoidance will play a mediating role in the improvement observed in the AIR + Mindfulness experimental group compared to the relaxation therapy control group.

## Methods

### Design

This protocol was developed according to the Standard Protocol Items: Recommendations for Interventional Trials (SPIRIT) [30], and it was listed on the ClinicalTrials.gov register on July 20, 2023 (NCT05956405). We designed a parallel group, single-blind, randomized controlled trial (RCT) with two treatment arms and a three-month follow-up. Evaluation will be made of the effectiveness of AIR + Mindfulness (intervention group) compared to relaxation health and wellness therapy (active control group) in patients with Long COVID. Both interventions are structurally similar, consisting of eight two-hour weekly sessions followed by three monthly two-hour sessions. Both groups will attend online workshops for both the AIR + Mindfulness intervention and relaxation therapy. Both groups will receive support materials online and by email. The Consolidated Standards of Reporting Trials (CONSORT) [31] will be applied.

### Setting and study sample

Potential participants will primarily be recruited through collaboration with the Long COVID Association of Aragon (Spain), and they will be required to meet the following inclusion/exclusion criteria. Inclusion criteria: (a) aged 18 years and over; (b) provision of signed informed consent; (c) diagnosis of Long COVID by primary care physicians. Exclusion criteria: (a) under 18 years of age; (b) any diagnosis of a disease that may affect the central nervous system or other psychiatric diagnoses or acute psychiatric illnesses (e.g., severe range of depression, substance dependence or abuse, history of schizophrenia or other psychotic disorders, eating disorders); (c) any medical, infectious or degenerative disease that may affect mood; presence of delusional ideas; and hallucinations consistent or not with mood and suicide risk.

### Sample size

According to a previous study, when use of the AIR + Mindfulness intervention was compared to a similar active control group (i.e., relaxation therapy) on a sample of fibromyalgia patients, it demonstrated an overall improvement in the severity of the disorder––measured using the Fibromyalgia Impact Questionnaire Revised [32], a tool that evaluates the effect of this condition on a person’s wellbeing and quality of life, considering pain levels, physical functioning, sleep quality, fatigue, mood, and the impact of the condition on daily activities––with a moderate effect size (d = 0.60) at post-intervention [22]. Assuming a similar effect size in terms of quality of life on Long COVID symptomatology at post-intervention, in order to detect this difference with an overall α level of 5% and a statistical power set at 80% in two-tailed tests, 42 patients per group are required. Given that a dropout rate of around 15% of the participants is expected [33], the sample size will be increased until the number of 50 participants is achieved per group at the beginning of the study.

### Recruitment

The recruitment of potential participants will be carried out in collaboration with the Long COVID Association of Aragon. The association will be responsible for disseminating the study through its own communication channels without sharing any member data. This dissemination will include informative sessions during the association’s activities and will provide essential information on the study, in addition to the contact details of the research team for those interested in participating. Patients who express interest via email will receive further information by means of a telephone or video call, through which a member of the research group will explain the primary objective of the study. The evaluation process will also be explained at this time. Evaluation, both for the baseline measurement and subsequent follow-up measurements (immediately after the intervention and three months later), will be conducted online. The survey will be hosted on SurveyMonkey®. Prior to starting the questionnaire, participants will have the opportunity to read the information sheet, informed consent form, as well as the privacy regulations and personal data handling policy of the website. Individuals who meet the inclusion criteria, agree to participate, and complete, sign, and submit the signed informed consent form will gain access to the evaluation and will be permitted to complete the initial assessment. This recruitment process will remain active until the required sample size is achieved.

### Randomization, allocation, and blinding

A research assistant will be responsible for assigning a unique code to each participant and communicating it via email. This research assistant will be the sole person with access to participants’ personal data, along with their respective codes and the group to which they are assigned once the allocation process is completed. This information will be securely stored in a password-protected digital file. A duplicate file will be created on a hard drive to which only the lead researcher will have access. Once the baseline surveys have been completed, subject allocation will be conducted by a different member of the research team, who will not be involved in any other study-related tasks. This allocation will be based on a computer-generated random sequence obtained from a reputable source (https://www.randomizer.org). Participants will be assigned to either of these two groups: (1) AIR + Mindfulness group; (2) Control group based on relaxation therapy. Participants and psychotherapists conducting the interventions will not be blinded to the intervention condition for practical reasons. However, blinding will be maintained during the initial assessment (pre-intervention). Additionally, the research assistant responsible for sharing the assessment survey link will be blinded, as will the expert conducting the statistical analysis.

### Data collection and monitoring

**Fig. 1:**
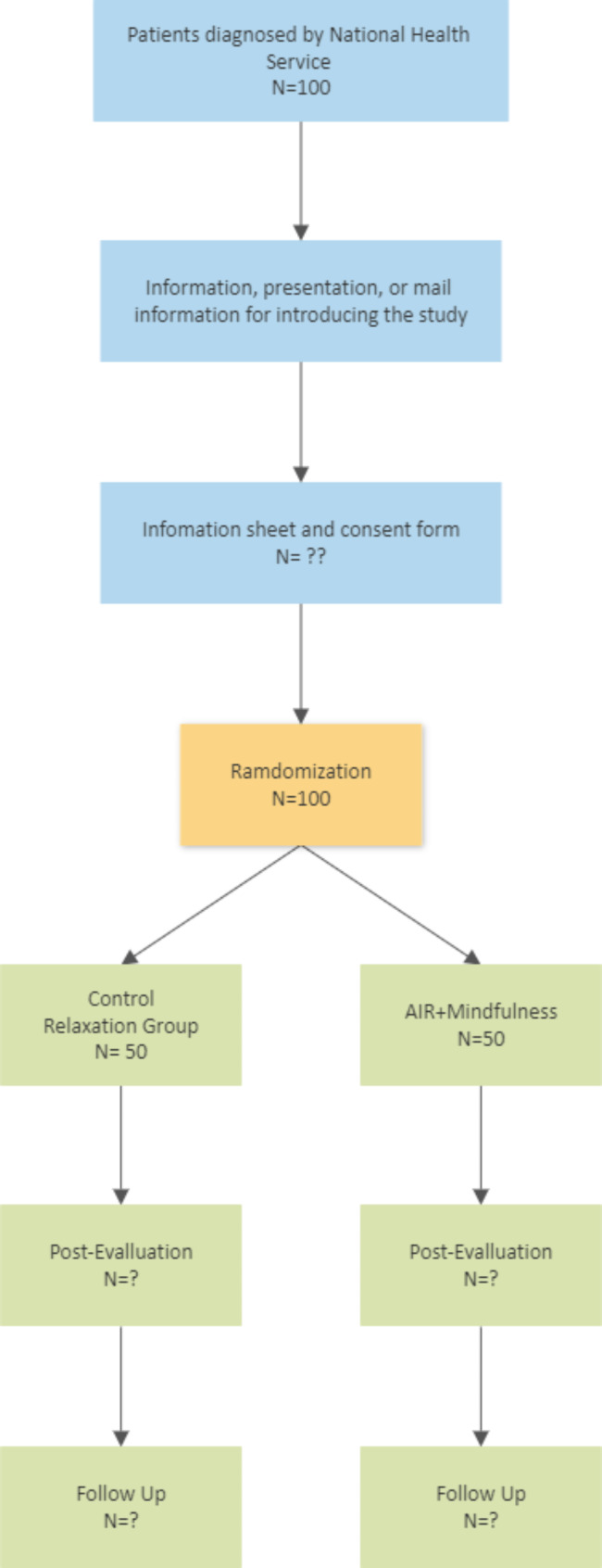
Flowchart for the randomized clinical trial

After completing the assigned intervention, participants will be contacted by the designated researcher responsible for monitoring and managing the evaluation processes. They will receive a link to an online survey to complete the post-treatment assessment and follow-up (at three months post-intervention). Throughout the study, there will be careful monitoring for any potential adverse effects experienced by participants although, owing to the nature of the intervention programs, such effects are not expected to occur [34]. Participants will be encouraged to promptly report any signs of worsening during sessions. However, should any adverse effects arise, the psychotherapist overseeing the intervention will report them to an independent data monitoring committee (IDMC) that is not directly involved in the study being monitored. This committee will comprise a biostatistician, a clinical psychologist, and a psychiatrist. They will assess the situation and determine if any additional measures are necessary to ensure the participant’s safety, such as referral to a hospital service, and the scientific integrity of the study. The anticipated RCT flowchart of participants in the study is provided as Fig. 1.

### Interventions

Both groups will undergo active interventions consisting of eight two-hour weekly sessions, followed by one session per month as booster over the following three months. Each session will be conducted online. Furthermore, participants will receive homework assignments and audio files with practice exercises for use between sessions. Prior to commencement of the program and participant allocation, an informative online session will be held. During this session, participants will have the chance to address any concerns they might have about the study. They will have received the study information sheet and the informed consent in advance.

#### AIR + mindfulness

This intervention will consist of an AIR protocol combined with elements of mindfulness training. The psychotherapeutic approach of the AIR program focuses on enhancing coping skills and strategies for dealing with fear and stressful situations. This is hypothetically achieved by interrupting the amygdala’s conditioned anxiety or fear responses. The program consists of psychological techniques including breathing exercises, meditation exercises, generative practices, compassion practices, cognitive behavioral therapy, and neuro-linguistic programming. AIR is a therapy that was specifically designed to alleviate symptoms in patients with CFS and fibromyalgia [15, 16, 18–20]. Neurobiology studies have demonstrated that exposure to acute stress stimuli, trauma, or extended periods of stressful expectations (such as those that can arise from enduring a pandemic or experiencing Long COVID), can result in the development of a conditioned response in the amygdala to new stimuli [35, 36]. Once conditioning becomes established in the amygdala, any neutral stimulus that occurs in the presence of this “trauma” acquires the ability to elicit fear in the organism. It triggers unconscious “fight and flight” responses, as if there were a real threat. This process is also associated with neuroinflammation of the brain in patients with conditions like CFS and fibromyalgia [3]. Thus, the stress conditioning process of the amygdala enters into a loop with all the abovementioned mechanisms perpetuating themselves over time [18, 19].

The retraining intervention focuses on dismantling fear conditioning in the amygdala, which contributes to the bodily symptoms observed in the disease. This is achieved by progressively undoing the conditioning and mitigating heightened levels of nervous system stimulation through the following methodology: (a) repeatedly disrupting the established unconscious conditioning; (b) substituting these fear and threat-related messages with messages of bodily safety; and (c) strengthening the activity of the prefrontal cortex and reducing the over-activation of the amygdala through mindfulness. The incorporation of safety messages results in a transformation of the unconscious implications of amygdala conditioning.

Mindfulness training of is associated with increased cortical thickness in the anterior insula (involved in interoception, affected by fibromyalgia and chronic fatigue) and the frontal cortex (involved in the integration of emotions as fear and cognition process). Moreover, this technique seems to change cognitive evaluations of what is considered a threat, decrease reflective thinking, and reduce stress. Mindfulness can also directly increase positive states of arousal [37–39]. Thus, the practice of mindfulness seems to reinforce the AIR intervention, given that the activity of the amygdala has been seen to decrease with its practice, as well as activation of the prefrontal cortex, an area also involved in diminishing the fear conditioning response.

Previous studies suggest that the practice of mindfulness causes effective and lasting results for the symptomatology and underlying pathology of post-viral fatigue, such as Long COVID. It has been found to reduce fatigue and unrefreshing sleep, and for those with post-viral infections, to improve immunity and reduce pathogen-driven inflammation [40, 41].

The intervention will be administered by a clinical psychologist who is trained in and has experience in facilitating groups for both the AIR program and mindfulness-based interventions. The content of each session is described in Table 1.

**Table 1:** AIR + Mindfulness program

Session 1	Overview of the retraining and mindfulness course. Introduction to the course. Goals. Work method. Developing a regular mindfulness practice. Mindfulness practices. Guidelines for the regulation of the nervous system. Thought diary. 3-minute mindfulness practice. Health visualization.
Session 2	Hypothesis on the origin of Long COVID. Theoretical aspects. The amygdala-insula-limbic hypothesis on how stress activates the nervous system. Conditioning and reconditioning, and health visualization. Breathing meditation.
Session 3	Main technique of reeducation of the amygdala and the insula. Reconditioning. Learning to break physical activations caused by stimuli and symptoms. Patterns, meanings, and unconscious negative emotions. Somatic responses in the body. Guidelines for establishing a daily practice. Body scan meditation.
Session 4	Attitudes with retraining: reducing stress and expectations during the intervention. Meanings and activation caused by the fear of failure in terms of recovery. Disidentification and acceptance of symptoms. Introduction to the work of subpersonalities. Awareness of personality traits. Mindfulness practice. Incorporation of informal practices into daily life. Walking meditation.
Session 5	Management of symptoms and related negative emotions. Disidentification and radical acceptance of symptoms. Resistance to symptoms. Lifestyle and pace to support retraining. How to pace yourself, sleep more soundly, expose yourself to daylight, healthy lifestyle and cope with crises. Smooth Flow Practice.
Session 6	The AIR accelerator technique. Retraining of repeated somatic signals, patterns, beliefs, behaviors related to persistent Covid disease. Disidentification of mental contents. Mindfulness practice. visual metaphors.
Session 7	Awareness of limiting beliefs related to the disease. How to identify and change them with tonsillar reeducation work. Motivation and meaning of life. Meditation on values.
Session 8	Cycles and stages of recovery and return to normal life. How to incorporate the tools into daily life as health improves. How to stay healthy in the long term. Positive visualizations of the future. Positive psychology. Exercise on gratitude. Cultivating positive emotions. Practice on Reconnecting with happiness. Protocol review and summary

#### Relaxation intervention

The program for the active control group includes progressive muscle relaxation training, as proposed by Bernstein and Borkovec [42], with the addition of visualization and breathing practices. The intervention will be administered by a clinical psychologist who is experienced with groups and is trained in relaxation therapy. The sessions contained in this program are described in Table 2.

**Table 2:** Relaxation program

Session 1	Presentation of the group and the relaxation objectives. Basic principles of progressive muscle relaxation. Brief explanation of the initial procedure with 16 muscle groups. Progressive muscle relaxation practice with 16 muscle groups. Identifying the sensations of and difficulties with relaxation.
Session 2	Sharing homework-related experiences and doubts. Imagination training and visualization techniques. Practice of progressive muscle relaxation with 16 muscle groups. Identifying the sensations of and difficulties with relaxation.
Session 3	Sharing homework-related experiences and doubts. Brief explanation of the procedure with 7 muscle groups. Practice of progressive muscle relaxation with 7 muscle groups. Visualization practice: orange. Identifying the sensations of and difficulties with relaxation.
Session 4	Sharing homework-related experiences and doubts. Practice of progressive muscle relaxation with 7 muscle groups. Visualization practice: the beach. Identifying the sensations of and difficulties with relaxation.
Session 5	Sharing homework-related experiences and doubts. Brief explanation of the procedure with 4 muscle groups. Practice of progressive muscle relaxation with 4 muscle groups. Visualization practice: the landscape. Visualization practices: the globe. Identifying the sensations of and difficulties with relaxation.
Session 6	Sharing homework-related experiences and doubts. Brief explanation of relaxation by evocation, relaxation by evocation + counting, and relaxation by counting. Relaxation by evocation. Mental relaxation and visualization practice: the perfect day. Identifying the sensations of and difficulties with relaxation.
Session 7	Sharing homework-related experiences and doubts. Explanation of relaxation techniques through breathing. Breathing practice: count, lengthen, hold. Visualization: the ray of light. Identifying the sensations of and difficulties with relaxation.
Session 8	Sharing homework-related experiences and doubts. Explanation: bringing the practice to my daily life, maintenance of habits. Breathing practice. Practice 4 muscle groups. Display. Identifying the sensations of and difficulties with relaxation.

### Outcomes

Data will be collected using a battery of self-reported questionnaires administered at baseline, after the intervention (post-intervention,) and at three-month follow-up. Outcomes of the AIR + Mindfulness and the relaxation conditions will be evaluated and compared. All variables and assessment periods of the study can be found in Table 3.

**Table 3:** Study Outcomes

Instrument	Assessment Area	Time
Bespoke survey	Sociodemographic	Baseline
SF-36	Quality of life	Baseline, post-treatment and 3-month follow-up
SCPGS	Pain intensity	Baseline, post-treatment and 3-month follow-up
PCS	Catastrophizing	Baseline, post-treatment and 3-month follow-up
MFIS	Fatigue	Baseline, post-treatment and 3-month follow-up
ISI	Insomnia	Baseline, post-treatment and 3-month follow-up
GAD-7	Anxiety	Baseline, post-treatment and 3-month follow-up
PHQ-9	Depression	Baseline, post-treatment and 3-month follow-up
MEF-30	Memory	Baseline, post-treatment and 3-month follow-up
FFMQ-24	Mindfulness	Baseline, post-treatment and 3-month follow-up
ERQ	Emotion Regulation	Baseline, post-treatment and 3-month follow-up
AAQ-II	Experiential avoidance	Baseline, post-treatment and 3-month follow-up

SF-36, Short Form Health Survey; SCPGS, Spanish Chronic Pain Grading Scale; PCS, Pain Catastrophizing Scale; MFIS, Modified Fatigue Impact Scale; ISI, Insomnia Severity Index; GAD-7, General Anxiety Disorder; PHQ-9, Patient Health Questionnaire; MEF-30, Memory Failures of Everyday; FFMQ-24, Five Facet Mindfulness Questionnaire; ERQ, Emotion Regulation Questionnaire; AAQ-II, Acceptance and Action Questionnaire-II.

#### Main outcome

The main outcome will be health-related quality of life measured with the Short Form-36 Health Survey (SF-36) [43, 44]. The SF-36 consists of 36 items that are scored on Likert-type scales from 1 to 3.5, or 6 depending on the specific item. It measures the following eight dimensions of health: physical function, physical role, aches and pains, general health, vitality, social function, emotional role, and mental health. In addition, the SF-36 incorporates a health progress item. For each dimension, the items are coded, aggregated, and transformed into a scale that ranges from 0 (the worst state of health for that dimension) to 100 (the best state of health). A score of 50 is typically considered the average or norm for the general population. The SF-36 general health (total) score will be considered the main outcome, and the SF-36 subscales described will be considered secondary outcomes. The Spanish version of the SF-36 has demonstrated good psychometric properties, establishing it as a reliable and valid tool for measuring health-related quality of life in the Spanish-speaking population. Internal consistency values for Cronbach’s alpha exceed 0.7 in all subscales as well as the total score [45].

#### Secondary outcomes

The intensity of chronic pain will be measured with the Spanish Chronic Pain Grading Scale (SCPGS) [46]. It comprises 10 items that are used to assess the intensity of pain at different times of the day, as well as its impact on the patient’s quality of life and daily functioning. Each item is scored on a scale from 0 to 10, where 0 means no pain, and 10 indicates the most intense pain imaginable. The total score ranges from 0 to 100, with a higher score indicating a greater intensity of chronic pain. The scale has been validated in Spanish patients with chronic pain and is considered a useful and reliable tool for the assessment of chronic pain in clinical practice and research (α = 0.87) [47].

Pain catastrophizing will be evaluated with the Pain Catastrophizing Scale (PCS) validated in Spanish [48]. This tool is a self-report questionnaire that measures catastrophized pain and consists of 13 items related to over-focusing on pain sensations, magnifying the threat value of pain sensations, and perceiving oneself as unable to control pain intensity. Items are rated in relation to their frequency of occurrence on a five-point scale, from never (0) to almost always [4]. The total PCS score is calculated as the algebraic sum of the scores for each item and ranges from 0 to 52, with higher scores reflecting a greater degree of pain catastrophizing. This version has adequate internal consistency (α = 0.79) and acceptable test-retest reliability (r = 0.84).

The Modified Fatigue Impact Scale (MFIS) is a multidimensional scale that has been used in numerous chronic pathologies [49]. It consists of 21 items distributed into three subscales: physical, cognitive, and psychosocial. Physical fatigue refers to the feeling of weakness and body exhaustion, accompanied by discomfort or even muscle pain and an inability to relax, which occurs after physical exertion; cognitive fatigue is a cognitive slowing or a feeling of dullness, which makes it difficult to perform tasks that require sustained mental effort; and psychosocial fatigue is the general feeling of exhaustion derived from carrying out social activities and outside the home. Each item is rated on a five-point Likert scale (from 0, “never,” to 4, “almost always”). The total score ranges from 0 to 84. A score of 38 has been established as a cut-off point to define the presence of fatigue. It assesses different aspects related to fatigue and cognitive and emotional affectation in a single form. It values physical and cognitive aspects, but is firmly focused on mental fatigue and its consequences, particularly the impact that fatigue has on the patient. The dimensions have shown adequate internal consistency (total score: α = 0.92; physical subscale: α = 0.88; cognitive subscale: α = 0.92, and psychosocial subscale: α = 0.65).

The Insomnia Severity Index (ISI) is a 7-item scale that assesses the symptoms of daytime and nocturnal insomnia and is widely used in the field of clinical research [50]. Each item is rated on a five-point Likert scale (e.g., 0 = no problem; 4 = very severe problem), yielding a total score ranging from 0 to 28. The information collected by this tool is related to the onset and maintenance of sleep, waking up in the morning, interference with daily functioning, deterioration perceived and attributed to the sleep problem, concerns about sleep problems, and satisfaction with sleep patterns. The validation of the Spanish version of the ISI has adequate internal consistency indices (Cronbach’s α = 0.82); it is significantly correlated with poor sleep quality, fatigue, anxiety, and depression; and it allows discrimination between people with good and bad sleep patterns [51].

Anxious symptomatology will be evaluated with the Generalized Anxiety Disorder-7 (GAD-7), which is made up of seven self-reported items [52]. Each item is scored on a four-point Likert scale (0 = “not at all”; 1 = “several days”; 2 = “more than half the days”; 3 = “nearly every day”), which results in a total score that can range from 0 to 21, with higher values reflecting more severe anxiety symptoms over the previous two weeks. Symptom severity can be organized into four categories: 0–4 (minimal anxiety), 5–9 (mild anxiety), 10–14 (moderate anxiety), and 15–21 (severe anxiety). The validation in Spanish has a sensitivity of 0.93 and a specificity of 0.85 [53].

The PHQ-9 is a nine-item self-report measure that assesses the presence of depressive symptoms based on the DSM-IV criteria for major depressive episode [54]. It refers to the symptoms experienced during the previous two weeks. The PHQ-9 is scored on a Likert scale (“never” = 0; “several days” = 1; “more than half the days” = 2; “almost every day” = 3), and the total score ranges from 0 to 27. Symptom severity can be organized into four categories: 0–4 (minimum), 5–9 (mild), 10–14 (moderate), 15–19 (moderate to severe), 20–27 (severe). The Spanish version of the scale will be used, which has demonstrated good psychometric properties (sensitivity = 87%; and specificity = 88%; Cronbach’s α = 0.835) [55].

The Memory Failures of Everyday Questionnaire (MFE) is one of the most widely used self-report questionnaires to assess daily forgetfulness [56]. Through 30 items and five response options (from 0 = “never” to 4 = “always”) memory failures and their frequency in daily life are evaluated. Several cut-off points are established: below 8 (optimal memory functioning), between 8 and 35 (normal functioning with memory lapses without influence on daily performance), between 36 and 50 (mnesic impairment with some impact on daily activity), and above 50 (moderate or severe memory impairment with greater impact on daily functioning). Studies have observed good internal consistency (α = 0.92).

#### Mechanistic measures

Mindfulness skills will be measured with a short version (24 items) of the Five Facet Mindfulness Questionnaire (FFMQ-24) [57]. This version is an adaptation of the original 39-item FFMQ, a questionnaire that measures the following five facets of mindfulness: observing (i.e., noticing internal and external experiences such as sensations, thoughts, or emotions), describing (i.e., labeling internal experiences with words), acting with awareness (i.e., focusing on one’s activities in the present moment as opposed to behaving automatically), nonjudging of inner experience (i.e., taking a nonevaluative stance toward thoughts and feelings), and nonreacting to inner experience (i.e., allowing thoughts and feelings to come and go, without getting caught up by them). Participants indicate the degree to which each of the items is generally true for them on a five-point Likert scale, from 1 (‘never or very rarely true’) to 5 (‘very often or always true’). Scores from the subscales can be summed to produce a total score that ranges between 24 and 120, with higher scores reflecting greater levels of mindfulness skills. The Spanish version of the short FFMQ-24 has good internal consistency values for the total score (α = 0.70), and subscales (α values ranging from 0.65 to 0.80) [58].

Emotional regulation capacity, which refers to an individual’s ability to modify the components of their emotional experience with respect to its frequency, form, duration, and intensity, will be measured with the Emotion Regulation Questionnaire (ERQ) [59]. This scale consists of 10 items, to which participants respond using a seven-point Likert scale (1 = “strongly disagree,” 7 = “strongly agree”). The ERQ is designed to measure the tendency of respondents to regulate their emotions through [1] cognitive reappraisal (6 items), and [2] expressive suppression (4 items). The cognitive reappraisal strategy modifies emotional reactions at the time of its gestation, managing to change the emotional experience. On the other hand, expressive suppression only modifies emotional expression and is an intentional hiding of the actual experience without altering it. The Spanish version of the ERQ shows adequate internal consistency (cognitive reappraisal: α = 0.89–0.90; expressive suppression: α = 0.76–0.80), test-retest reliability, and convergent/discriminant validity [60].

Experiential avoidance will be measured with the Acceptance and Action Questionnaire-II (AAQ-II) [61]. As opposed to psychological flexibility, experiential avoidance is a psychological construct associated with poorer mental health and reflects the tendency to avoid or escape from internal emotional experiences, thoughts, memories, sensations, or bodily sensations that individuals perceive as distressing, uncomfortable, or unwanted. It usually involves engaging in behaviors intended to distract or distance oneself from these experiences. It comprises seven items scored on a seven-point Likert scale, and whose total score is calculated by adding all the items. Higher scores mean greater experiential avoidance. The Spanish version of the AAQ-II presents good internal consistency (α = 0.84) and test-retest reliability (r = 0.79) [62].

## Data analysis plan

The distribution of the sociodemographic and clinical variables will be presented by frequencies (percentages), medians (interquartile range, IQR) or means (standard deviations, SD), according to their level of measurement and statistical distribution.

The primary analysis will consist of a comparison between AIR + Mindfulness (intervention arm) and relaxation therapy (control arm) at post-intervention, considering the quality-of-life main outcome (SF-36) as a continuous variable. It will be performed using a repeated measures design on an intention-to-treat (ITT) basis, and a linear mixed effects regression by means of the restricted maximum likelihood (RML) method, controlling for age and sex. The “treatment by time” interaction will be calculated to determine possible differences between the study arms over time. The slope coefficient, representing the adjusted mean difference change, as well as its 95% confidence interval (95% CI), will be calculated. Hedge’s g statistic, as an effect size measure of between-group differences, will be calculated from the raw data. The rule of thumb for effect size interpretation will be 0.20 = small, 0.50 = medium, and 0.80 = large.

Secondary analysis will explore the effects achieved in the primary outcome at three-month follow-up, as well as in the secondary outcomes at post-intervention and at three-month follow-up, following the same analytical approach. Sensitivity analysis will assess the effects of missing data, which will be assumed to be missing at random and replaced by multiple imputations based on chained equations, including the main and secondary outcomes of all the waves––as well as routinely collected sociodemographic data. Other secondary analyses will explore reliable change and clinically significant change in quality-of-life improvements using the Jacobson and Truax method [63]. These classifications will be used to calculate the absolute risk reduction (ARR) and the number needed to treat (NNT); a 95% CI for each NNT will also be calculated. We will use a two-sided test with a 0.05 significance level. No corrections for multiple comparisons will be made for the primary analysis––because only one contrast will be made. Secondary analyses will be corrected for multiple comparisons by adjusting the significance threshold according to the Benjamini-Hochberg procedure [64].

The potential role of mindfulness skills, emotion regulation, and experiential avoidance as potential mediators of improvements in both the primary and secondary outcomes will be explored. For this purpose, (a) pre-follow-up differential scores for primary and secondary outcomes will be considered dependent variables; (b) pre-post differential scores for mindfulness skills, emotion regulation, and experiential avoidance will be calculated and included as process variables; (c) the group condition (AIR + Mindfulness vs. relaxation therapy) will be considered the independent variable. The mediating analyses will be conducted using ordinary least squares (OLS) regression and path analysis for mediating analyses involving continuous dependent variables. Standardized regression coefficients of bootstrapped indirect effects will be estimated, as well as their 95% CIs based on 10,000 bootstrapped samples, considering a significant mediating effect when the 95% CI of the indirect effect does not include zero.

## Discussion

Long COVID syndrome is a clinical condition characterized by the persistence of symptoms for at least 12 weeks after the onset of COVID-19. While its manifestation can vary greatly in terms of intensity and frequency, some of the most common symptoms include respiratory problems, myalgia, extreme fatigue, moodiness, cognitive impairment, and difficulty sleeping [65, 66]. This study protocol presents the design of an RCT with the primary objective of evaluating the effectiveness of an AIR + Mindfulness intervention, in comparison to a relaxation therapy program, for enhancing the health-related quality of life in patients diagnosed with Long COVID. Given the global impact of this syndrome, there has been a notable increase in scientific interest for the development and evaluation of different intervention strategies aimed at improving the health status of these patients. Indeed, certain psychological interventions, such as AIR and mindfulness have demonstrated positive outcomes in alleviating psychological distress for Long COVID [26]. However, the evidence remains limited, and further research is necessary to replicate and expand upon these findings [28, 29].

Despite the promising results to date, there is currently no medical and/or psychological treatment with sufficient empirical evidence that enables effective management of the symptoms of Long COVID syndrome [10, 67]. Given that scientific research suggests that the symptoms of Long COVID are due, albeit partially, to immune dysregulation and chronic inflammation in the body [1, 3], the hypothesis presented in this paper is that the AIR + Mindfulness program will effectively benefit these patients. This belief is grounded in its demonstrated efficacy in conditions of a comparable nature, such as fibromyalgia and CFS, by influencing processes that impact the immune system, such as reducing levels of C-reactive protein and brain-derived neurotrophic factor, which are considered a key element in a variety of neuroplasticity processes such as pain modulation, nociception, and hyperalgesia [9, 13, 22, 68].

The study has several strengths. First, this is the first RCT to be conducted in Spain that applies a psychotherapy program for the management of symptoms derived from Long COVID. Secondly, owing to the little scientific evidence currently available in the field, the results obtained will be of great value for the process of designing and developing effective intervention programs to manage this syndrome. Third, in addition to the post-treatment evaluation, a follow-up will be carried out three months after the end of the intervention program, which will allow its medium-term effects to be evaluated. The fact that the intervention is delivered in a group format can generate a context in which the participants’ involvement is promoted, favoring greater adherence and, consequently, greater benefits. Finally, an active control group will be used, as recommended in research using contemplative programs [69], as it allows possible changes in the variables to be attributed to the intervention and not only to other factors, such as the attention shown by the instructor or the relationship with the group.

With regard to the potential limitations of the study, it should be noted that the intervention will be conducted solely in the city of Zaragoza (northeastern Spain), without the possibility of extending the study area. Therefore, the generalizability of the results will be limited. Other potential difficulties may be that since patient participation in the study will be voluntary, there could be a higher dropout rate due to unforeseen life circumstances or health-related conditions. Finally, all the variables evaluated will be collected only through self-report questionnaires; thus, all data collected will have the limitations of this particular methodology.

## Data Availability

In accordance with the International Committee of Medical Journal Editors (ICMJE), the data generated by this trial will be made available upon reasonable request to researchers (i) who provide a methodologically sound proposal and (ii) whose proposed use of the data has been approved by an independent ethical review committee. The data sharing plan includes all anonymized and fully deidentified participant data collected during the trial, as well as the study protocol, statistical analysis plan, and data dictionary with descriptive labels. Data will become available following each publication with no end date and for any analytical purpose that is related to achieving aims in the original approved proposal. The database will be encrypted and password protected. Passwords will be provided by the corresponding author to interested researchers who meet both conditions (i) and (ii).

## Abbreviations

AAQ-II: Acceptance and Action Questionnaire-II
AIR: Amygdala & insula retraining
CFS: Chronic fatigue syndrome
ERQ: Emotion Regulation Questionnaire
FFMQ-24: Five Facet Mindfulness Questionnaire
GAD-7: General Anxiety Disorder
ISI: Insomnia Severity Index
ME: Myalgic encephalomyelitis
MEF-30: Memory Failures of Everyday
MFIS: Modified Fatigue Impact Scale
PCS: Pain Catastrophizing Scale
PHQ-9: Patient Health Questionnaire
RCT: Randomized controlled trial
SCPGS: Spanish Chronic Pain Grading Scale
SF-36: Short Form Health Survey

## References

[1] Ortona E, Malorni W, Long COVID. To investigate immunological mechanisms and sex/gender related aspects as fundamental steps for a tailored therapy. Eur Respir J. 2022;59(2). 10.1183/13993003.02245-2021 10.1183/13993003.02245-2021 PMC8462012 34531277

[2] de Jong CMM, Le YNJ, Boon GJAM, Barco S, Klok FA, Siegerink B (2023). Eight lessons from 2 years of use of the Post-COVID-19 functional status scale. Eur Respir J.

[3] Wong TL, Weitzer DJ. Long COVID and myalgic encephalomyelitis/chronic Fatigue Syndrome (ME/CFS)-A systemic review and comparison of clinical presentation and symptomatology. Med. 2021;57(5). 10.3390/medicina57050418 PMC8145228 33925784

[4] Guidelines NICE. COVID-19 rapid guideline: managing the long-term effects of COVID-19. NICE Guidel. 2020;(18 December 2020):1–35. Available from: https://www.nice.org.uk/terms-and

[5] Sykes DL, Holdsworth L, Jawad N, Gunasekera P, Morice AH, Crooks MG, Post. -COVID-19 Symptom Burden: What is Long-COVID and How Should We Manage It? Lung. 2021;199(2):113–9. 10.1007/s00408-021-00423-z 10.1007/s00408-021-00423-z PMC7875681 33569660

[6] Bucciarelli V, Nasi M, Bianco F, Seferovic J, Ivkovic V, Gallina S et al. Depression pandemic and cardiovascular risk in the COVID-19 era and long COVID syndrome: Gender makes a difference. Trends Cardiovasc Med. 2022;32(1):12–7. Available from: https://linkinghub.elsevier.com/retrieve/pii/S1050173821001158 10.1016/j.tcm.2021.09.009 PMC8490128 34619336

[7] Bautista-Rodriguez E, Cortés-Álvarez NY, Vuelvas-Olmos CR, Reyes-Meza V, González-López T, Flores-delosÁngeles C (2023). Stress, anxiety, depression and long COVID symptoms. Fatigue Biomed Heal Behav.

[8] Fancourt D, Steptoe A, Bu F (2023). Psychological consequences of long COVID: comparing trajectories of depressive and anxiety symptoms before and after contracting SARS-CoV-2 between matched long- and short-COVID groups. Br J Psychiatry.

[9] Fernández-de-las-Peñas C, Florencio LL, Gómez-Mayordomo V, Cuadrado ML, Palacios-Ceña D, Raveendran AV (2021). Proposed integrative model for post-COVID symptoms. Diabetes Metab Syndr Clin Res Rev.

[10] Han JH, Womack KN, Tenforde MW, Files DC, Gibbs KW, Shapiro NI (2022). Associations between persistent symptoms after mild COVID-19 and long-term health status, quality of life, and psychological distress. Influenza Other Respi Viruses.

[11] Varatharaj A, Thomas N, Ellul MA, Davies NWS, Pollak TA, Tenorio EL et al. Neurological and neuropsychiatric complications of COVID-19 in 153 patients: a UK-wide surveillance study. The Lancet Psychiatry. 2020;7(10):875–82. Available from: https://linkinghub.elsevier.com/retrieve/pii/S221503662030287X 10.1016/S2215-0366(20)30287-X PMC7316461 32593341

[12] Davis HE, Assaf GS, McCorkell L, Wei H, Low RJ, Re’em Y (2021). Characterizing long COVID in an international cohort: 7 months of symptoms and their impact. eClinicalMedicine.

[13] Scordo KA, Richmond MM, Munro N. Post–COVID-19 Syndrome: Theoretical Basis, Identification, and Management. AACN Adv Crit Care. 2021;32(2):188–94. Available from: https://aacnjournals.org/aacnacconline/article/32/2/188/31445/Post-COVID-19-Syndrome-Theoretical-Basis 10.4037/aacnacc2021492 33942071

[14] Andrés-Rodríguez L, Borràs X, Feliu-Soler A, Pérez-Aranda A, Rozadilla-Sacanell A, Montero-Marin J et al. Immune-inflammatory pathways and clinical changes in fibromyalgia patients treated with Mindfulness-Based Stress Reduction (MBSR): A randomized, controlled clinical trial. Brain Behav Immun. 2019;80(December 2018):109–19. 10.1016/j.bbi.2019.02.030 10.1016/j.bbi.2019.02.030 30818032

[15] Cash E, Salmon P, Weissbecker I, Rebholz WN, Bayley-Veloso R, Zimmaro LA et al. Mindfulness meditation alleviates fibromyalgia symptoms in women: results of a randomized clinical trial. Ann Behav Med. 2015;49(3):319–30. Available from: https://www.embase.com/search/results?subaction=viewrecord&id=L607906153&from=export. 10.1007/s12160-014-9665-0 PMC4802162 25425224

[16] Perez-Aranda A, Feliu-Soler A, Montero-Marin J, Garcia-Campayo J, Andres-Rodriguez L, Borras X (2019). A randomized controlled efficacy trial of mindfulness-based stress reduction compared with an active control group and usual care for fibromyalgia: the EUDAIMON study. Pain.

[17] Quirk GJ, Garcia R, González-Lima F (2006). Prefrontal mechanisms in extinction of conditioned fear. Biol Psychiatry.

[18] Gupta A (2010). Can amygdala retraining techniques improve the wellbeing of patients with Chronic Fatigue Syndrome. J Holist Healthc.

[19] Gupta A (2002). Unconscious amygdalar fear conditioning in a subset of Chronic Fatigue Syndrome patients. Med Hypotheses.

[20] Toussaint LL, Whipple MO, Abboud LL, Vincent A, Wahner-Roedler DL. A mind-body technique for symptoms related to fibromyalgia and chronic fatigue. Explor J Sci Heal. 2012;8(2):92–8. 10.1016/j.explore.2011.12.003 10.1016/j.explore.2011.12.003 22385563

[21] Gotink RA, Meijboom R, Vernooij MW, Smits M, Hunink MGM. 8-week Mindfulness Based Stress Reduction induces brain changes similar to traditional long-term meditation practice – A systematic review. Brain Cogn. 2016;108:32–41. 10.1016/j.bandc.2016.07.001 10.1016/j.bandc.2016.07.001 27429096

[22] Sanabria-Mazo JP, Montero-Marin J, Feliu-Soler A, Gasión V, Navarro-Gil M, Morillo-Sarto H et al. Mindfulness-based program plus amygdala and insula retraining (MAIR) for the treatment of women with fibromyalgia: A pilot randomized controlled trial. J Clin Med. 2020;9(10):1–16. Available from: https://www.embase.com/search/results?subaction=viewrecord&id=L2005203889&from=export. 10.3390/jcm9103246 PMC7599726 33050630

[23] Mayorga NA, Manning KF, Garey L, Viana AG, Ditre JW, Zvolensky MJ. The Role of Experiential Avoidance in Terms of Fatigue and Pain During COVID-19 Among Latinx Adults. Cognit Ther Res. 2022;46(3):470–9. 10.1007/s10608-022-10292-2 10.1007/s10608-022-10292-2 PMC8802248 35125558

[24] Brenning K, Waterschoot J, Dieleman L, Morbée S, Vermote B, Soenens B et al. The role of emotion regulation in mental health during the COVID-19 outbreak: a 10-wave longitudinal study. Stress Heal. 2022;(September). 10.1002/smi.3204 PMC9874444 36252954

[25] Ryan RM, Deci EL. Self-determination theory: Basic Psychological needs in motivation, Development, and Wellness. Guilford Publications; 2017. p. 756.

[26] Toussaint LL, Bratty AJ. Amygdala and Insula Retraining (AIR) Significantly Reduces Fatigue and Increases Energy in People with Long COVID. Zahiruddin S, editor. Evidence-Based Complement Altern Med. 2023;2023:1–8. Available from: https://www.hindawi.com/journals/ecam/2023/7068326/10.1155/2023/7068326 PMC10365910 37492483

[27] Ceban F, Leber A, Jawad MY, Yu M, Lui LMW, Subramaniapillai M et al. Registered clinical trials investigating treatment of long COVID: a scoping review and recommendations for research. Infect Dis (Auckl). 2022;54(7):467–77. 10.1080/23744235.2022.2043560 10.1080/23744235.2022.2043560 PMC8935463 35282780

[28] Hawke LD, Nguyen ATP, Ski CF, Thompson DR, Ma C, Castle D (2022). Interventions for mental health, cognition, and psychological wellbeing in long COVID: a systematic review of registered trials. Psychol Med.

[29] Al-Jabr H, Hawke LD, Thompson DR, Clifton A, Shenton M, Castle DJ et al. Interventions to support mental health in people with long COVID: a scoping review. BMC Public Health. 2023;23(1):1–18. 10.1186/s12889-023-16079-8 10.1186/s12889-023-16079-8 PMC10280822 37340400

[30] Chan A, Tetzlaff JM, Altman DG. 2013 Statement: Defining Standard Protocol Items for Clinical Trials. Ann Intern Med. 2016;158(3):200–7. 10.7326/0003-4819-158-3-201302050-00583 PMC5114123 23295957

[31] Schulz KF, Altman DG, Moher D. CONSORT 2010 Statement: updated guidelines for reporting parallel group randomised trials. BMC Med. 2010;8(18). 10.1186/1741-7015-8-18 PMC2860339 20334633

[32] Bennett RM, Friend R, Jones KD, Ward R, Han BK, Ross RL (2009). The revised fibromyalgia impact questionnaire (FIQR): validation and psychometric properties. Arthritis Res Ther.

[33] Montero-Marin J, Navarro-Gil M, Puebla-Guedea M, Luciano JV, Van Gordon W, Shonin E et al. Efficacy of ‘Attachment-Based Compassion Therapy’ in the treatment of Fibromyalgia: a Randomized Controlled Trial. Front PSYCHIATRY. 2018;8. 10.3389/fpsyt.2017.00307 PMC5775966 29387020

[34] Baer R, Crane C, Montero-Marin J, Phillips A, Taylor L, Tickell A (2021). Frequency of self-reported unpleasant events and harm in a mindfulness-based program in two General Population samples. Mindfulness (N Y).

[35] Sotres-Bayon F, Cain CK, LeDoux JE (2006). Brain mechanisms of fear extinction: historical perspectives on the contribution of Prefrontal Cortex. Biol Psychiatry.

[36] Johansen JP, Tarpley JW, LeDoux JE, Blair HT. Neural substrates for expectation-modulated fear learning in the amygdala and periaqueductal gray. Nat Neurosci. 2010;13(8):979–86. Available from: https://www.nature.com/articles/nn.2594 10.1038/nn.2594 PMC2910797 20601946

[37] Goldin PR, Gross JJ. Effects of mindfulness-based stress reduction (MBSR) on emotion regulation in social anxiety disorder. Emotion. 2010;10(1):83–91. Available from: http://www.ncbi.nlm.nih.gov/pubmed/20141305 10.1037/a0018441 PMC4203918 20141305

[38] Hölzel BK, Carmody J, Evans KC, Hoge EA, Dusek JA, Morgan L (2009). Stress reduction correlates with structural changes in the amygdala. Soc Cogn Affect Neurosci.

[39] Kral TRA, Schuyler BS, Mumford JA, Rosenkranz MA, Lutz A, Davidson RJ. Impact of short- and long-term mindfulness meditation training on amygdala reactivity to emotional stimuli. Neuroimage. 2018;181(1):301–13. Available from: https://linkinghub.elsevier.com/retrieve/pii/S1053811918306256 10.1016/j.neuroimage.2018.07.013 PMC6671286 29990584

[40] Porter N, Jason LA (2022). Mindfulness Meditation interventions for Long COVID: Biobehavioral Gene expression and Neuroimmune Functioning. Neuropsychiatr Dis Treat.

[41] Natarajan A, Shetty A, Delanerolle G, Zeng Y, Zhang Y, Raymont V et al. A systematic review and meta-analysis of long COVID symptoms. Syst Rev. 2023;12(1):1–19. 10.1186/s13643-023-02250-0 10.1186/s13643-023-02250-0 PMC10220332 37245047

[42] Bernstein DA, Borkovec TD. Progressive relaxation training: a manual for the helping professions. Research Press.; 1973. p. 80.

[43] Alonso J, Prieto L, Antó J (1995). The Spanish version of the SF-36 Health Survey (the SF-36 health questionnaire): an instrument for measuring clinical results. Med Clin.

[44] Alonso J, Prieto L, Ferrer M, Vilagut G, Broquetas JM, Roca J (1998). Testing the measurement properties of the Spanish version of the SF-36 Health Survey among male patients with Chronic Obstructive Pulmonary Disease. J Clin Epidemiol.

[45] Vilagut G, Ferrer M, Rajmil L, Rebollo P, Permanyer-Miralda G, Quintana JM et al. The Spanish version of the Short Form 36 Health Survey: a decade of experience and new developments. Gac Sanit. 2005;19(2):135–50. 10.1157/13074369 10.1157/13074369 15860162

[46] Ruiz-López R, Pagerols M, Collado A. Cuestionario Del dolor en español: resultados de Su Empleo Sistematizado Durante El Periodo 1990-93. Pain. 1993;11(6).

[47] Ferrer-Peña R, Gil-Martínez A, Pardo-Montero J, Jiménez-Penick V, Gallego-Izquierdo T, La Touche R. Adaptación y validación de la Escala de gradación del dolor crónico al español. Reumatol Clin. 2016;12(3):130–8. Available from: https://linkinghub.elsevier.com/retrieve/pii/S1699258X15001205 10.1016/j.reuma.2015.07.004 26298083

[48] García Campayo J, Rodero B, Alda M, Sobradiel N, Montero J, Moreno S. Validación de la versión española de la escala de la catastrofización ante el dolor (Pain Catastrophizing Scale) en la fibromialgia. Med Clin (Barc). 2008;131(13):487–92. 10.1157/13127277 10.1157/13127277 19007576

[49] Kos D, Kerckhofs E, Carrea I, Verza R, Ramos M, Jansa J (2005). Evaluation of the modified fatigue impact scale in four different European countries. Mult Scler.

[50] Morin CM, Belleville G, Bélanger L, Ivers H (2011). The insomnia severity index: psychometric indicators to detect insomnia cases and evaluate treatment response. Sleep.

[51] Fernandez-Mendoza J, Rodriguez-Muñoz A, Vela-Bueno A, Olavarrieta-Bernardino S, Calhoun SL, Bixler EO et al. The Spanish version of the Insomnia Severity Index: A confirmatory factor analysis. Sleep Med. 2012;13(2):207–10. 10.1016/j.sleep.2011.06.019 10.1016/j.sleep.2011.06.019 22172961

[52] Spitzer R, Kroenke K, Williams J, Löwe B (2006). A brief measure for assessing generalized anxiety disorder: the GAD-7. Arch Intern Med.

[53] García-Campayo J, Zamorano E, Ruíz MA, Pardo A, Freire O, Pérez-Páramo M (2009). Cultural Adaptation into Spanish of the generalized anxiety disorder Scale-7 (GAD-7) scale. Eur Psychiatry.

[54] Kroenke K, Spitzer RL, Williams JBW (2001). The PHQ-9: validity of a brief depression severity measure. J Gen Intern Med.

[55] Diez-Quevedo C, Rangil T, Sanchez-Planell L, Kroenke K, Spitzer RL. Validation and Utility of the Patient Health Questionnaire in Diagnosing Mental Disorders in 1003 General Hospital Spanish Inpatients. Psychosom Med. 2001;686(63):679–86. Available from: https://journals.lww.com/psychosomaticmedicine/Abstract/2001/07000/Validation_and_Utility_of_the_Patient_Health.21.aspxhttps://citeseerx.ist.psu.edu/viewdoc/download?doi=10.1.1.540.3655&rep=rep1&type=pdf 10.1097/00006842-200107000-00021 11485122

[56] Lozoya Delgado P, Ruiz Sánchez de León JM, Pedrero Pérez EJ (2012). Validación De Un cuestionario de quejas cognitivas para adultos jóvenes: relación entre las quejas subjetivas de memoria, la sintomatología prefrontal y El estrés percibido. Rev Neurol.

[57] Bohlmeijer E, Klooster PM, Fledderus M, Veehof M, Baer R (2011). Psychometric properties of the five facet mindfulness questionnaire in depressed adults and development of a short form. Assessment.

[58] Asensio-Martínez Á, Masluk B, Montero-Marin J, Olivan-Blázquez B, Navarro-Gil MT, García-Campayo J (2019). Validation of five facets Mindfulness Questionnaire – Short form, in Spanish, general health care services patients sample: prediction of depression through mindfulness scale. PLoS ONE.

[59] Gross JJ, John OP (2003). Individual differences in two emotion regulation processes: implications for Affect, relationships, and well-being. J Pers Soc Psychol.

[60] Cabello R, Salguero JM, Fernández-Berrocal P, Gross JJ (2013). A Spanish adaptation of the emotion regulation questionnaire. Eur J Psychol Assess.

[61] Bond FW, Hayes SC, Baer RA, Carpenter KM, Guenole N, Orcutt HK (2011). Preliminary Psychometric properties of the Acceptance and Action Questionnaire-II: a revised measure of psychological inflexibility and experiential avoidance. Behav Ther.

[62] Ruiz FJ, Langer Herrera ÁI, Luciano C, Cangas AJ, Beltrán I (2013). Measuring experiential avoidance and psychological inflexibility: the Spanish version of the Acceptance and Action Questionnaire-II. Psicothema.

[63] Jacobson NS, Truax P. Clinical significance: A statistical approach to defining meaningful change in psychotherapy research. J Consult Clin Psychol. 1991;59(1):12–9. Available from: http://doi.apa.org/getdoi.cfm?doi=10.1037/0022-006X.59.1.12. 10.1037//0022-006x.59.1.12 2002127

[64] Benjamini Y, Yekutieli D. The control of the false discovery rate in multiple testing under dependency. Ann Stat. 2001;29(4):1165–88. Available from: https://projecteuclid.org/journals/annals-of-statistics/volume-29/issue-4/The-control-of-the-false-discovery-rate-in-multiple-testing/10.1214/aos/1013699998.full

[65] Di Toro A, Bozzani A, Tavazzi G, Urtis M, Giuliani L, Pizzoccheri R (2021). Long COVID: long-term effects?. Eur Hear Journal Suppl.

[66] Veronese N, Bonica R, Cotugno S, Tulone O, Camporeale M, Smith L (2022). Interventions for improving long COVID-19 symptomatology: a systematic review. Viruses.

[67] Schrimpf A, Braesigk A, Lippmann S, Bleckwenn M. Management and treatment of long COVID symptoms in general practices: an online-based survey. Front Public Heal. 2022;10. 10.3389/fpubh.2022.937100 PMC9513068 36176520

[68] Montero-Marin J, Andrés-Rodríguez L, Tops M, Luciano JV, Navarro-Gil M, Feliu-Soler A et al. Effects of attachment-based compassion therapy (ABCT) on brain-derived neurotrophic factor and low-grade inflammation among fibromyalgia patients: A randomized controlled trial. Sci Rep. 2019;9(1):15639. Available from: https://www.embase.com/search/results?subaction=viewrecord&id=L629732772&from=export. 10.1038/s41598-019-52260-z PMC6821772 31666651

[69] Ma SH, Teasdale JD. Mindfulness-based cognitive therapy for depression: replication and exploration of differential relapse prevention effects. J Consult Clin Psychol. 2004;72(1):31–40. Available from: http://www.embase.com/search/results?subaction=viewrecord&from=export&id=L38380527 10.1037/0022-006X.72.1.31 14756612

